# Supplementary figures and images for: Automation of Infectious Focus Assay for Determination of Filovirus Titers and Direct Comparison to Plaque and TCID50 Assays
Source: Microorganisms. 2021 Jan 12;9(1):156. doi: 10.3390/microorganisms9010156 (PMC7826780; doi:10.3390/microorganisms9010156)

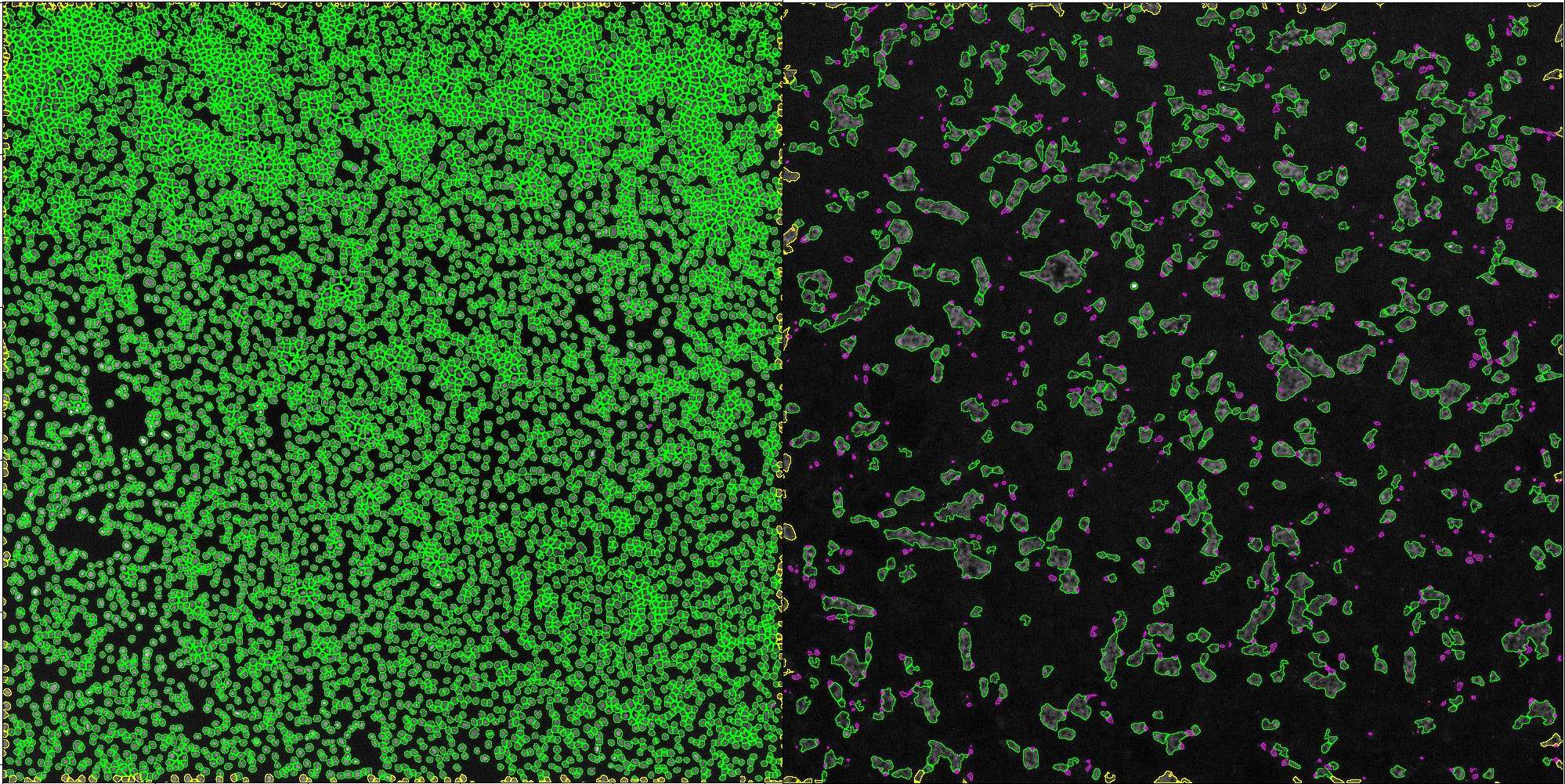

Supplement: Supplementary file 1 [file microorganisms-09-00156-s001.zip › microorganisms-1064524-supplementary-published (final)/Figure S1.tif]
